# Proposal for the creation of a national strategy for precision medicine in cancer: a position statement of SEOM, SEAP, and SEFH

**DOI:** 10.1007/s12094-017-1740-0

**Published:** 2017-08-31

**Authors:** P. Garrido, A. Aldaz, R. Vera, M. A. Calleja, E. de Álava, M. Martín, X. Matías-Guiu, J. Palacios

**Affiliations:** 1 Medical Oncology, Ramón y Cajal University Hospital, IRYCIS, University of Alcalá, Madrid, Spain; 2 Pharmacy, Clínica Universidad de Navarra, Working Group PkGen from the SEFH, Pamplona, Spain; 3 Pharmacy, Virgen de Macarena University Hospital, Seville, Spain; 4 Pathology, Virgen del Rocío University Hospital, Institute of Biomedicine of Seville (IBiS)/CSIC/University of Sevilla, Seville, Spain; 5 Centro de Investigación Biomédica en Red de Oncología, CIBERONC-ISCIII, Madrid, Spain; 6 Medical Oncology, Instituto de Investigación Sanitaria Gregorio Marañón, Universidad Complutense, Madrid, Spain; 7 Pathology and Molecular Genetics, Hospital Universitari de Bellvitge, IDIBELL Hospital Universitari Arnau de Vilanova, University of Lleida, IRBLLEIDA, Lleida, Spain; 8 Pathology, Ramón y Cajal University Hospital, IRYCIS, University of Alcalá, Madrid, Spain; 9 Medical Oncology, Complejo Hospitalario de Navarra, Pamplona, Spain

**Keywords:** Oncology, Precision medicine, Consensus

## Abstract

Precision medicine is an emerging approach for disease treatment and prevention that takes into account individual variability in genes, environment, and lifestyle for each person. Precision medicine is transforming clinical and biomedical research, as well as health care itself from a conceptual, as well as a methodological viewpoint, providing extraordinary opportunities to improve public health and lower the costs of the healthcare system. However, the implementation of precision medicine poses ethical–legal, regulatory, organizational, and knowledge-related challenges. Without a national strategy, precision medicine, which will be implemented one way or another, could take place without the appropriate planning that can guarantee technical quality, equal access of all citizens to the best practices, violating the rights of patients and professionals, and jeopardizing the solvency of the healthcare system. With this paper from the Spanish Societies of Medical Oncology, Pathology, and Hospital Pharmacy, we highlight the need to institute a consensual national strategy for the development of precision medicine in our country, review the national and international context, comment on the opportunities and challenges for implementing precision medicine, and outline the objectives of a national strategy on precision medicine in cancer.

## Rationale

With this paper from the Spanish Societies of Medical Oncology (SEOM), Pathology (SEAP), and Hospital Pharmacy (SEFH), we propose to highlight the need to institute a consensual national strategy for the development of precision medicine in our country and promote its implementation in the clinical practice with equity and with assurances in terms of quality, efficiency, and legal guarantee, in addition to contributing to the sustainability of the healthcare system.

## Introduction

There is no universal definition for the term “precision medicine”, although probably, the most widely accepted is the one provided by the US National Institutes of Health (NIH) that defines it as “an emerging approach for disease treatment and prevention that takes into account individual variability in genes, environment, and lifestyle for each person” [1]. Though at times used interchangeably with personalized medicine, the National Research Council prefers the term precision medicine instead, because personalized medicine might suggest that the different strategies for treatment and prevention are developed solely for each individual to the extent that “individual” refers to the specific individual and not in terms of belonging to a particular biotype, as is truly the case with precision medicine.

This definition gives rise to two fundamental consequences. On the one hand, it entails a change of paradigm in medicine in that the approach to disease is founded on the genetic and molecular bases of health and disease to estimate risks and inform decisions regarding prevention, diagnosis, and treatment. Illnesses previously considered as a single disease have now been disaggregated into several entities, with different causal mechanisms that call for different strategies. Inversely, diseases that were regarded as disparate must be approached from the same point of view, given that they have molecular mechanisms in common.

The concept of precision medicine necessarily involves modifying the point at which health care commences, understanding that patients’ treatment and needs depend more on their particular characteristics than the generic name of the disease. Likewise, knowing which nosological groups a person is predisposed to as an individual will make it possible to develop better prevention strategies. Furthermore, individuals take a more active role in their own health, by understanding their natural predisposition to suffer certain diseases.

Precision medicine has already become a reality in daily clinical practice in certain disciplines such as oncology; not only is its implementation an ethical mandate and obligation of policy insofar as it represents an indisputable improvement in the treatment of patients and prevention of disease, but it has also been proven to foster the sustainability of the healthcare system in certain cases, by selecting patients with a greater likelihood of response, keeping patients from being exposed to expensive, unnecessary treatments, while minimizing complications derived from therapies with scant or no possibility of response, and enables the most efficient preventive actions to be selected for each individual.

## International and national contexts and initiatives

By means of several national plans, the United States, United Kingdom, France, Germany, China, and other developed countries such as Finland and Estonia have implemented national strategies endowed with state funding to mobilize and strengthen the industry and technological development associated with precision medicine, channel the necessary private and public resources to put them into effect, improve infrastructure, and increase the current applications of this kind of medicine.

Internationally, the International Consortium for Personalised Medicine (ICPerMed) constitutes the most relevant project in Europe. It comprises the European Commission and more than 30 European and extra-European partners and funding agencies [2]. Its main objective is to stimulate research and the implementation of precision medicine by means of meetings, workshops, conferences, surveys, strategic publications, and joint initiatives. It origin lies in the preparatory workshops organized by the European Commission, together with several subsequent initiatives, including the seventh Framework Program and the establishment of EuroBioForum and the consortium of CASyM (http://www.casym.eu) in 2011 [3].

In France, for example, the Institut National du Cancer (INCa) has an institutional framework to incorporate precision medicine into standard health care and the France Médecine Génomique Plan 2025, published in 2016 and with a projection until 2025, seeks to equip this country with the means and industrial structure it needs to introduce this new approach into health care and for this discipline to be placed as a driver of economic development in France [4]. Other countries, such as Estonia, Iceland, or the United Kingdom, have developed initiatives to create population biobanks that make it possible to establish associations between biomarkers, clinical history, and lifestyle.

In the United States, the Precision Medicine Initiative, announced by then President Obama, allocated 216 million dollars in the 2016 fiscal year to fund a shared initiative of the NIH, the National Cancer Institute (NCI), the Food and Drug Administration (FDA), and the Office of the National Coordinator for Health Information Technology (ONC). The most distinctive aspect of this project is the creation of a database in which one million volunteers will provide genetic data, biological samples, and clinical information with the aim of predicting risk, understanding how and why common diseases occur and to improve diagnostic and treatment strategies [5].

To date, a general strategy on precision medicine has not been developed at state level in Spain. Different National Strategies compile recommendations for the development of precision medicine (for instance, the National Healthcare System’s Strategies in Cancer and Rare Diseases). In addition, there are national initiatives, such as the one put forth by the National Institute of Health Carlos III [call for projects in the field of precision medicine, participation in the Network for Excellency for Research and Innovation on Exosomes (REDIEX) or a European intergovernmental organization for lifescience resources (ELIXIR), etc.], together with local projects, such as the Comprehensive Plan for Genomic Medicine in Catalonia, the Medical Genome Project in Andalusia, the Future Clinic project in the Valencian Region, or the MEDEA project in Extremadura [6].

## Opportunities

Precision medicine is a reality in practical clinical health care and has begun to shift the paradigms in medicine and even change how diseases are classified. In general, precision medicine enhances effectiveness and efficiency, since it makes it possible for the most appropriate strategies to be used for each patient on the basis of the molecular mechanism underlying the illness and the individual’s genetic variability. Furthermore, it fosters the application of the most suitable therapeutic scheme for patients, given that it factors in the genetic variability determining drug metabolism and pharmacodynamics, together with the environmental factors that also play out in their disposition. Thus, it prevents exposing patients to drugs that are not useful for them, decreasing the possibilities of adverse events related to drugs with no possibility of response, as well as the secondary complications derived from treating patients with ineffective drugs and the loss of opportunity that this entails.

From an economic perspective, precision medicine is regarded as an opportunity to develop an industrial sector of high strategic, healthcare, scientific, and economic value. The incorporation of our country at an early stage would provide us with the chance to be technologically independent in a sector that is more and more necessary, and would also enable us to export knowledge and technology in a new industrial sector. All this represents an unprecedented economic opportunity in our country that would call for a hefty investment and mobilization of all parties involved to achieve innovative technological solutions (that include the fields of industry and information technology) and a new form of economic development that enables the device to be sustained beyond its start-up and to respond to several technological challenges, in particular to the development of the necessary information technology capacities. Precision medicine is subject to international competition and our country should not remain on the side lines.

Finally, precision medicine is proposed as a tool to contribute to rationalizing healthcare expenditure and to the sustainability of the healthcare system; generating the data needed to generalize initiatives that demonstrate cost effectiveness.

## Challenges

### Ethical–legal and regulatory challenges

With the development of precision medicine, citizens and healthcare systems confront new challenges, such as that of maintaining the balance between risks and benefits, bearing in mind the unprecedented ethical, economic, social, and legal implications, in particular with respect to data protection.

The identification of biomarkers and massive sequencing techniques is based on the collection and analysis of a tremendous amount of information (“big data”). In this context, it is imperative that the confidentiality of sensitive personal data be guaranteed, especially in multicenter, multinational projects that call for the shared use of data, but also within the context of biobanks (despite the fact that donors are generally anonymous, some biobanks require that donors can be identified).

On the other hand, if samples are used for subsequent research, doubts may arise as to who actually holds the property rights over the samples, the validity of the consent that was given, or about the right to the information (or to non-information). This occurs to the degree to which genetic testing makes it possible to identify or confirm the mutations responsible for a disease that will probably develop in the future or to identify a predisposition to developing it when there are as yet no preventive techniques or effective treatments available for many of those genetic diseases. Clear regulations are, therefore, fundamental with the aim of guaranteeing that the principle of universal and equal access to healthcare be guaranteed. Furthermore, it is paramount that the risk of citizens being excluded on the basis of their genetic data and their predisposition to suffer certain diseases be approached both legally and ethically.

The ethical dimension is an integral part of the implementation of an initiative of this nature. We must be able to respond to the ethical and legal issues that arise from the consent provided by citizens for the use of their health data and to the complications derived from data anonymization, the management of secondary discoveries and of untoward incidents.

### Organizational and knowledge-related challenges

From a purely instrumental point of view, information technology systems must be put into place to enable the management and sharing of the vast amount of data generated by means of new generation sequencing techniques [Big Data and Information and Communications Technologies (ITC) solutions]. The transformation to precision medicine calls for new professional roles that are as yet not included in healthcare systems, thus requiring the involvement of professionals in the field of bioinformatics and other professionals that currently participate under the umbrella of research.

Moreover, clinical information and data from complementary testing are currently safeguarded by the healthcare profession and/or by the institution that performed the specific determinations; however, this model is not suitable for managing data coming from the next generation of sequencing and that could have future medical applications for individuals or for their descendants.

From a scientific perspective, precision medicine calls for an even greater understanding of the molecular bases of disease and of the interaction between genes and the environment.

Furthermore, studies must be initiated to evaluate the implementation of healthcare applications, which requires substantial investment and a multidisciplinary approach.

Likewise, genetic, pharmacogenetic, and “omic” sciences must be bolstered at the undergraduate and postgraduate levels of training; promoting the continuous education offer involving the leading scientific societies and establishing the accreditation of reference centers. Pharmacogenomics is one part of precision medicine, but it must be complemented with pharmacology in order for this new discipline to be created.

Even more important are the challenges associated with the paucity of knowledge the general population has regarding precision medicine. The acceptance of treatment or prevention recommendations can be difficult, especially bearing in mind that they deal with complex concepts and that the dissemination of mistaken ideas about a kind of genetic determinism without factoring in the modulating effect of lifestyle and environment, could lead to a feeling of defeatism and helplessness or, in contrast, could lead to society’s becoming “medicalized”, and instigate the performance of unnecessary or dangerous testing, as well as to potentially mistaken decisions about reproduction.

In this regard, the creation of a national platform is decisive within the overall project for the incorporation of precision medicine that can analyze and control the translation of scientific findings to clinical practice.

Finally, we must be mindful of the fact that in this context, every new drug or technique can be developed for a relatively small proportion of patients, making it necessary for there to be other models to facilitate access to the market from a regulatory perspective.

## Objectives of a national strategy on precision medicine

Taking into account the benefits, as well as the challenges derived from this area of medicine, we set forth the following objectives of the different areas as being a priority:

Healthcare and quality objectives:

- To raise awareness among the population and decision makers about the importance of precision medicine in current medical and healthcare practice and its projections for the future.
- To incorporate precision medicine into the strategic plans already in force and to prioritize it in national health and research strategies, giving it the importance that scientific evidence confers upon it in each case uniformly throughout the territory.
- To facilitate access to precision medicine to all patients with cancer and individuals with rare and common diseases that are susceptible to it by creating sequencing platforms that are capable of covering the whole country.
- To guarantee that the techniques used are accurate and reliable, assuring the use of the best scientific evidence available in basic and translational research.
- To facilitate the generation of information about health outcomes.
- To develop the national legal framework to make the regulation from the European Parliament and the European Council regarding personal data protection and the free circulation data feasible as well as to develop those aspects left to national legislation.

Area of knowledge:

- To accelerate the design and execution of genetics-based studies, exploring the basic aspects of tumor biology and setting up a network of knowledge about oncology that generates data and enables it to be shared to stimulate information technology and scientific discoveries and to inform clinical decisions.
- From a regulatory perspective, to promote the performance of academic studies on response prediction by means of biomarkers in those areas not covered by industry.
- To foster the availability of comprehensive databases that are validated and accessible and that include genomic, biomedical, clinical, and lifestyle information. To stimulate networking through corporate structures, enhancing the shared use and access to data regarding subpopulations, generated both within the framework of clinical trials, as well as in real life.
- To organize technology that will make it possible to analyze starting up a national center that enables the genetic data generated to be processed and used and to offer services for current applications.
- To develop training for healthcare professionals in the application of precision medicine in healthcare practice.
- To promote studies that analyze how pharmacogenomics and pharmacokinetics relate to one another and how they relate to measures of health outcomes.
- To stimulate university-level training in this new branch of knowledge and to develop the new competences and technology needed to respond to the challenge of using and interpreting data on a large scale.
- To guarantee training of regulatory agents with the aim of maintaining the necessary regulatory structure that can guarantee innovation and make it possible to protect public health.
- To promote citizens’ knowledge about health and disease by encouraging the participation of volunteers.

Efficiency and sustainability:

- To put our country among the nations having the capacity to develop and apply precision medicine and, therefore, to export knowledge and technology.
- To develop the necessary regulatory frameworks to guarantee a system to evaluate new drugs, biomarkers, and efficient diagnostic methods.
- To foster a long-term economic model, capable of integrating and developing the industrial fabric needed to sustain the incorporation of precision medicine into health on a large scale.
- To create an observatory that can monitor the evolution of this field of medicine in its medical, technological, ethical, and regulatory dimensions.
- To prepare the legal regulations and code of ethics that make it possible to respond to the ethical and legal demands associated with the collection, conservation, and treatment of clinical and genomic data.

## Conclusion

Precision medicine is transforming clinical and biomedical research, as well as health care itself from a conceptual, as well as a methodological viewpoint, providing extraordinary opportunities to improve public health and lower the costs of the healthcare system. Without a national strategy, precision medicine, which will be implemented one way or another, could take place without the appropriate planning that can guarantee technical quality, equal access of all citizens to the best practices, violating the rights of patients and professionals, and jeopardizing the solvency of the healthcare system.
